# Ketogenic Diet Increases Serum and White Adipose Tissue SIRT1 Expression in Mice

**DOI:** 10.3390/ijms232415860

**Published:** 2022-12-13

**Authors:** Rossella Tozzi, Federica Campolo, Enke Baldini, Mary Anna Venneri, Carla Lubrano, Salvatore Ulisse, Lucio Gnessi, Stefania Mariani

**Affiliations:** 1 Department of Molecular Medicine, “Sapienza” University of Rome, 00161 Rome, Italy; 2 Department of Experimental Medicine, Section of Medical Physiopathology, Food Science and Endocrinology, “Sapienza” University of Rome, Viale del Policlinico 155, 00161 Rome, Italy; 3 Department of Surgical Sciences, “Sapienza” University of Rome, 00161 Rome, Italy

**Keywords:** SIRT1, ketogenic diet, ketone bodies, obesity, epigenetic regulators, adipose tissue

## Abstract

Overnutrition and its sequelae have become a global concern due to the increasing incidence of obesity and insulin resistance. A ketogenic diet (KD) is widely used as a dietary treatment for metabolic disorders. Sirtuin1 (SIRT1), a metabolic sensor which regulates fat homeostasis, is modulated by dietary interventions. However, the influence of nutritional ketosis on SIRT1 is still debated. We examined the effect of KD on adipose tissue, liver, and serum levels of SIRT1 in mice. Adult C57BL/6J male mice were randomly assigned to two isocaloric dietary groups and fed with either high-fat KD or normal chow (NC) for 4 weeks. Serum SIRT1, beta-hydroxybutyrate (βHB), glucose, and triglyceride levels, as well as SIRT1 expression in visceral (VAT), subcutaneous (SAT), and brown (BAT) adipose tissues, and in the liver, were measured. KD-fed mice showed an increase in serum βHB in parallel with serum SIRT1 (r = 0.732, *p* = 0.0156), and increased SIRT1 protein expression in SAT and VAT. SIRT1 levels remained unchanged in BAT and in the liver, which developed steatosis. Normal glycemia and triglycerides were observed. Under a KD, serum and white fat phenotypes show higher SIRT1, suggesting that one of the molecular mechanisms underlying a KD’s potential benefits on metabolic health involves a synergistic interaction with SIRT1.

## 1. Introduction

Fat excess, adiposopathy, and metabolic disorders are increasing exponentially all over the world [1,2]. Ketogenesis is a metabolic process involved in controlling energy homeostasis by crucial epigenetic mechanisms [3,4]. Ketone bodies (KBs), namely acetoacetate, beta-hydroxybutyrate (βHB), and acetone, produced in the liver when fatty acids are broken down for energy, are used as an alternative metabolic fuel in case of nutrient deprivation, such as starvation and extended caloric restriction (CR). Non-canonical and multi-dimensional roles of KBs in signaling and therapy are currently known [5]. Alterations evoked by endogenous or exogenous ketosis [6] improve anti-inflammatory and mitochondrial activities, mnemonic and muscle performance, and reduce the incidence of tumors in middle-aged mice—ultimately improving the general health of the animals, with a net effect on the increase in life expectancy [7,8]. The ketogenic diet (KD) is effective also in the management of human metabolic diseases [9,10,11,12]. However, despite its widespread use, the precise role and underlying molecular mechanisms by which KD achieves clinical effects are not fully understood. A common occurrence happening during ketogenesis, as well as during CR, is the increase in intracellular concentrations of NAD+, a cofactor required to support the catalytic activity of enzymes called sirtuins (SIRTs) [13].

SIRTs are nicotinamide-adenine-dinucleotide (NAD+)-dependent cellular sensors associated with the control of mitochondrial energy metabolism, inflammation, genome stability, and autophagy through functions intrinsically linked to longevity [14,15]. Among the seven conserved class III histone deacetylases, SIRT1 is the most studied of the family. It efficiently allows cellular adaptation to CR with consequent metabolic benefits [16,17] but its activity is impaired in the case of overnutrition or increased energy availability [18,19,20]. As for KBs, the role of SIRT1 in the regulation of metabolic homeostasis is carried out through the epigenetic control of food intake, the regulation of the glyco-lipidic profile, and the differentiation of adipocytes and the mobilization of fat, thus protecting against diseases such as atherosclerosis [21], hepatic steatosis [22,23], diabetes mellitus [24,25], and osteoporosis [26,27]. Accordingly, such diseases are developed in SIRT1-knock-out mice and antagonized in SIRT1-transgenic mice [28].

Recent advances in understanding the outcomes shared by KBs and SIRT1 suggest joint work involving cooperative interaction between these players, with the same beneficial intent [3,29]. However, although experimental and clinical evidence reports the efficacy and safety of a KD, its mechanisms of action need to be further elucidated. The relationship between KBs and SIRT1, still non-conclusive, places SIRT1 among the candidates for the downstream effect of KBs.

Our hypothesis is that beneficial metabolic effects of ketosis involve SIRT1 adipose tissue. We investigated the effect of a low-carbohydrate, high-fat KD on SIRT1 levels in serum and tissues that play a prominent role in energy homeostasis, i.e., the adipose tissue, and the liver.

## 2. Results

### 2.1. KD Affects Food Intake without Affecting Body Weight and the Percentage of Body Fat

**Figure 1:**
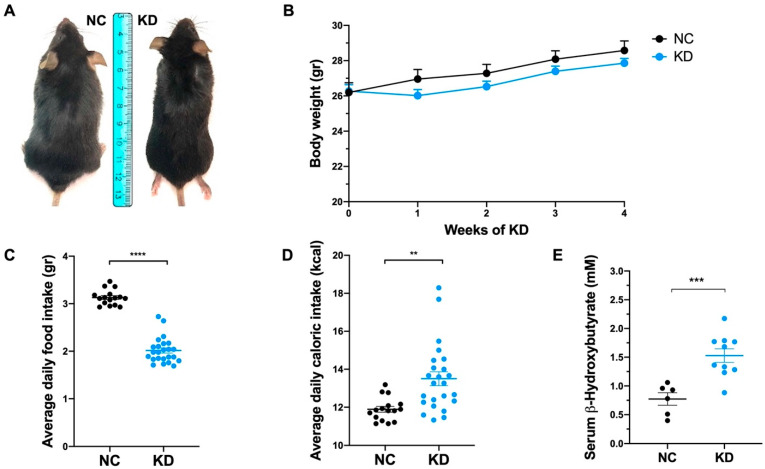
Ketogenic diet effects on body weight and food intake. (**A**) Representative photographs of mice under normal chow (NC) and ketogenic diet (KD) at the end of dietary treatment. (**B**) Body weight analysis during dietary treatment showing weight gain (g) of mice under NC (n = 8) and KD (n = 10). Data are expressed as mean ± SEM. (**C**) Scatter dot plot showing average daily food intake (g) of mice under NC and KD. Data are expressed as mean ± SEM. **** *p* < 0.001. (**D**) Scatter dot plot showing average daily caloric intake (kcal) of mice under NC and KD. Data are expressed as mean ± SEM. ** *p* < 0.01. (**E**) Dot plot showing βHB concentration (mM) in serum from mice under NC (n = 6) and KD (n = 10) at the end of dietary treatment. Data are expressed as mean ± SEM. *** *p* < 0.005.

To evaluate the effects of ketosis on SIRT1 levels we administered a high-fat diet to prevent low-carbohydrate-dependent weight loss. KD-fed mice immediately adapted to the new diet, and showed a transient body weight stabilization during the first week (Figure 1A,B). Despite KD-fed mice displaying a reduced daily food intake (Figure 1C), they gained the same amount of weight as control mice for the entire duration of the experiment, as demonstrated by similar weight trajectories (Figure 1B). The mean daily caloric intake (Figure 1D) was 1.7-fold higher in KD-fed mice than NC-fed mice. This difference is attributable to the fact that KD-fed mice can compensate for the higher energy content of the KD by their lower intake (Figure 1C), resulting in unaffected body fat distribution and weight.

As demonstrated by the measurement of serum βHB levels, ketosis was achieved and βHB concentration was significantly increased in KD-fed mice compared to NC-fed mice at the end of dietary treatment (Figure 1E).

**Figure 2:**
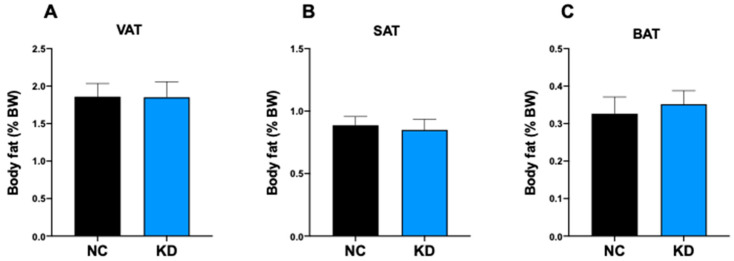
Ketogenic diet effects on fat mass. Analysis of fat coefficient (g fat/g body × 100) of: (**A**) perigonadal VAT, (**B**) SAT, and (**C**) interscapular BAT of mice under normal chow (NC, n = 6) and ketogenic diet (KD, n = 10) at the end of dietary treatment. Data are expressed as mean ± SEM.

To evaluate the effect of ketosis on fat mass we determined the fat coefficient of the adipose tissue depots in KD-fed mice and NC-fed mice. This analysis revealed that 4-week ketosis is not able to trigger any change in adipose tissue depot weight, either white visceral adipose tissue (VAT) (Figure 2A), white subcutaneous adipose tissue (SAT) (Figure 2B) or brown interscapular adipose tissue (BAT) (Figure 2C).

### 2.2. Ketosis Increases Serum and Adipose Tissue Levels of SIRT1

To explore the response of SIRT1 to nutritional ketosis, and to prevent the modulation of SIRT1 induced by weight loss, we fed animals with a low-carbohydrate, high-fat, KD.

**Figure 3:**
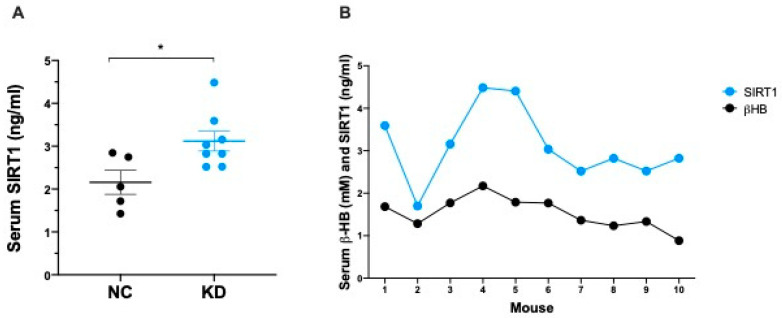
(**A**) Dot plot showing SIRT1 concentration (ng/mL) in serum from mice under NC (n = 5) and KD (n = 8) at the end of dietary treatment. Data are expressed as mean ± SEM. * *p* < 0.05. (**B**) Pearson correlation analysis between serum βHB and SIRT1 levels in mice under KD (n = 10). r = 0.732, *p* = 0.0156.

The administration of a 4-week KD resulted in a significant increase in the serum concentration of SIRT1 (Figure 3A). Circulating βHB (NC 0.773 ± 0.108 mM vs. KD 1.528 ± 0.117 mM) and SIRT1 (NC 2.158 ± 0.278 ng/mL vs. KD 3.119 ± 0.231 ng/mL) increases were consistent, and showed a high coefficient of correlation (r = 0.732, *p* = 0.0156) (Figure 3B).

**Figure 4:**
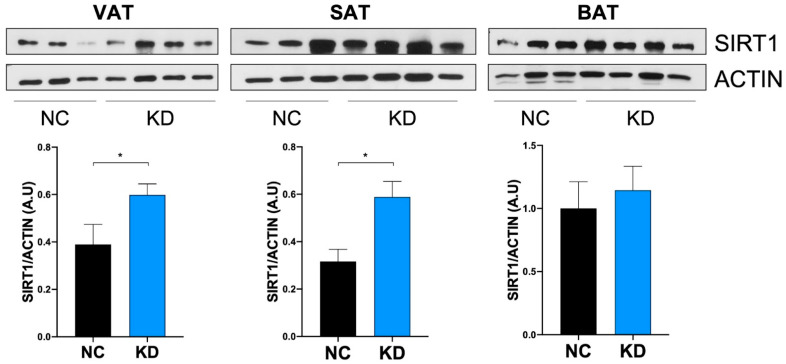
Ketogenic diet effect on adipose tissue levels of SIRT1. Western Blotting analysis of SIRT1 expression on visceral adipose tissue (VAT), subcutaneous adipose tissue (SAT), and interscapular brown adipose tissue (BAT) extracts from mice under normal chow (NC, n = 6) and ketogenic diet (KD, n = 10) at the end of dietary treatment. Densitometric analyses show protein expression levels expressed as arbitrary units ± SEM. * *p*  <  0.05.

Adipose tissue represents the central metabolic organ regulating whole body energy homeostasis. To examine the effect of ketosis on SIRT1 levels in adipose tissue, we analyzed SIRT1 expression in fat depots of KD-fed mice. As shown in Figure 4, SIRT1 protein was significantly increased in VAT and SAT of KD-fed mice compared to NC-fed mice, while SIRT1 did not appear significantly modulated by KD in BAT.

### 2.3. High-Fat KD Does Not Modulate SIRT1 in Liver

**Figure 5:**
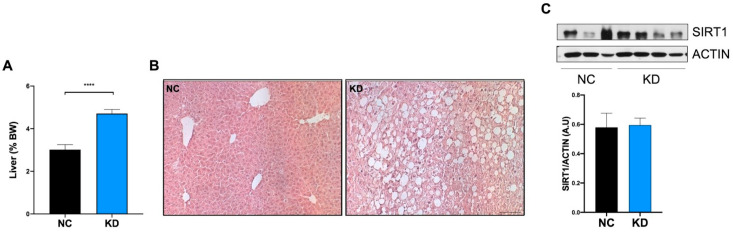
KD treatment effect on liver. (**A**) Analysis of liver coefficient (g liver/g body × 100) of mice under normal chow (NC, n = 6) and ketogenic diet (KD, n = 10) at the end of dietary treatment. Data are expressed as mean ± SEM. **** *p* < 0.001. (**B**) Representative images of liver morphology by Hematoxylin and Eosin staining on sections obtained from mice under NC and KD at the end of dietary treatment. Magnification 10×. Scale bar = 50 micron. (**C**) Ketogenic diet effect on liver levels of SIRT1. Western Blotting analysis of SIRT1 expression on liver extracts from mice under normal chow (NC, n = 6) and ketogenic diet (KD, n = 10) at the end of dietary treatment. Densitometric analyses show protein expression levels expressed as arbitrary units ± SEM.

The liver plays a pivotal role in the regulation of substrate availability, and adapts its metabolic profile weekly in response to nutrients. It stores excess nutrients and exports fat during fasting, metabolizes fatty acids into ketones, and responds to metabolic consequences of a high-fat KD. As expected, the administration of a high-fat KD resulted in hepatomegaly (Figure 5A) and liver steatosis (Figure 5B), characterized by hepatocyte ballooning, and increase in lipid droplets altering the canonical histology of the liver in the KD fed mice, compared to NC fed mice. At the molecular level, liver steatosis was not associated with an increase in SIRT1 protein (Figure 5C).

### 2.4. KD Controls the Glyco-Lipidic Profile

**Figure 6:**
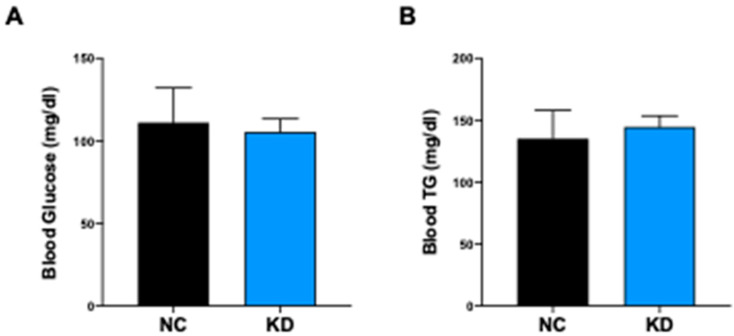
Ketogenic diet effect on blood levels of glucose and triglycerides. (**A**) Bar plot showing serum fasting glucose concentration (mg/dL) from mice under NC (n = 8) and KD (n = 10) at the end of dietary treatment. (**B**) Bar plot showing triglycerides (TG) serum concentration (mg/dL) from mice under NC (n = 8) and KD (n = 10) at the end of dietary treatment. Data are expressed as mean ± SEM.

The administration of high-fat diet regimens to C57BL/6J mice triggers hyperglycemia starting from the third week of dietary treatment [30]. To assess if KD could exert a protective role in preventing HFD-induced hyperglycemia, we measured serum glucose levels in KD-fed mice and NC-fed mice at the end of dietary treatment. Serum fasting glucose and triglycerides were in the normal range in KD-fed mice, indicating a positive role of βHB and SIRT1 in maintaining a normal glyco-lipid profile (Figure 6A,B) despite the high intake of lipids.

## 3. Discussion

In the present study, we evaluated the response to a 4-week high-fat KD on C57BL/6J mice to investigate whether nutritional ketosis exerts its effects through the downstream activation of SIRT1. We found that the increase in serum levels of βHB parallels a significant increase in SIRT1 circulating levels, and that a KD upregulates SIRT1 protein expression in white but not in brown adipose tissue, highlighting a different fat response to the KD-mediated SIRT1 metabolic effects. Furthermore, liver steatosis associated with a high-fat KD was not accompanied by modulation in intrahepatic SIRT1.

Adiposopathy due to fat excess and metabolic disorders, such as obesity and diabetes, is exponentially increasing all over the world [1,2], thus leading researchers to focus on new natural or technologically modified nutritional approaches for the treatment of overweight and its complications [31,32,33]. This has brought new insights into the pathophysiology of energy balance and weight disorders, as well as their possible means of evaluation.

Energy metabolism and food intake respond to fine interactions between hormones, cellular sensors, and nutraceutical regulators acting on adipose tissue, which is not an inert tissue but an organ essential in the regulation of whole-body metabolism. It is capable of determining metabolic flexibility by its fat storing capacity, thermoregulatory role, and adipokine production [34,35]. The variation in fat amount is one of the main drivers of SIRT1 activity. In line with this assumption, we recently found that SIRT1 shows a continuous, inverse pattern of expression which follows the whole spectrum of adiposity, from anorexia nervosa to obesity [20], and that blood SIRT1 assumes a trend consistent with that of circulating adipokines such as adiponectin and leptin [36]. SIRT1 is a nutrient-sensing (NAD)-dependent deacetylase associated with the response to calorie restriction (CR) and weight loss, in these conditions increased SIRT1 levels in rodents and humans have been observed [13,37,38,39]. Interestingly, SIRT1 reduces VAT deposition in animal models of obesity, and fat cell hypertrophy occurs when SIRT1 mRNA expression is low [40]. This phenotype is observed, also, in humans, in which the reduced expression of SIRT1 enhances the differentiation capacity of VAT-derived stem cells, fostering VAT expansion [41,42], and the reduction of SIRT1 mRNA transcription in VAT is associated with obesity [43].

As for SIRT1, the physiological role of KBs summarizes some aspects of CR. βHB, the predominant KB, represents the alternative fuel provision during starvation, acts as a signaling molecule, and shows a significant metabolic and therapeutic value associated with chronobiological [44] and anti-aging [45] functions. Interestingly, the metabolic shift towards fat oxidation and ketogenesis, during starvation or KD, is associated with initial mitochondrial stress characterized by increased levels of reactive oxygen species, and increased ratios of NAD+/NADH. In response to local inflammation, a protective and hormetic adaptation is carried out via SIRT1 and AMPK activation, and the consequences of the initial moderate metabolic stress include the upregulation of antioxidative and anti-inflammatory activities, and improved mitochondrial function [46,47]. AMPK, in turn, has the ability to increase NAD, and thus the activity of SIRT1 [48]. The close cooperation between AMPK and SIRT1 has been observed in hepatocytes [49], muscle cells [50], and adipocytes as well. Indeed, several compounds with insulin-sensitizing and lipolytic effects appear to activate the AMPK-SIRT1 pathway in 3T3L1 adipocytes [51,52,53], and specific adipocyte knock out for SIRT1 reduces the AMPK-mediated antioxidant and anti-inflammatory response in HFD rats [53]. Therefore, ketosis could rescue adipose tissue from inflammation also through the SIRT1-involved signaling pathway, and the interaction between AMPK.

In addition to the adaptive response to ketosis, KBs actively modulate metabolism via the epigenetic pathway. Epigenomic adaptation to food intake is mediated by the epigenetic role of both βHB and SIRT1. Via adipose tissue-specific signals involving PPARs, PGC1-α, mTORC1, p53, FOXO3a, AMPK, UCP1 etc., they intervene on DNA methylation, acetylation/deacetylation, histone post-translational modifications, and noncoding RNA regulation [3]. Moreover, recent studies reported the association of histone lysine β-hydroxybutyrylation with the pathogenesis of metabolic diseases [54], and showed that 3-hydroxybutyrate is involved in metabolic regulation of liver gene expression [51], and induces the expression of adiponectin in adipocytes [55].

Nutritional ketosis is currently extensively studied due to its effects on metabolic health. However, little is known regarding the mechanisms underlying the pleiotropic effects of KBs, despite evidence of clinical benefits exerted in animals [7,8] and humans [9,11,56,57,58,59].

We found that SIRT1 protein expression is upregulated in mouse white adipose tissue (WAT) under KD. Several studies have shown that adipose tissue responds to energy shortage with specific activation of SIRT1 [60], and that patients with severe obesity who experienced weight loss have a significant induction of SIRT1 expression in SAT [61]. However, with SIRT1 being an energy sensor sensitive to energy imbalance, we evaluated SIRT1 expression without weight loss influence. This was possible because mice can compensate for the higher energy content of the administered diet by reducing their intake [62] and, among mouse strains, C57BL6 mice particularly exhibit the most pronounced compensatory decreases in food intake with increases in the percentage of kilocalories consumed [63]. The upregulation of SIRT1 in WAT in the absence of weight loss may be attributed to ketosis. This finding is in line with the observation that the administration of a high-fat non-KD leads to the reduction of the SIRT1 gene and protein expression, in both WAT and BAT [60].

KD did not induce SIRT1 modulation in BAT. Nevertheless, different characteristics in fat depots play specific roles, reinforcing the paradigm that white and brown adipose tissue have pathognomonic properties and biological functions [35,64,65]. Indeed, SIRT1 activity in WAT should be distinct from that in BAT, and be considered independent [66,67]. Specifically, SIRT1 is required for the trans-differentiation of white preadipocytes [68,69], and acts mainly by limiting fat expandability via repression of PPAR𝛾 in WAT [13,70]. Accordingly, SIRT1-null mice showed reduction in uncoupling protein 1 (UCP1) in white SAT, without showing differences in BAT, supporting the essential activity of SIRT1 for WAT browning [66]. Intriguingly, the brown adipocytes in SIRT1-transgenic mice accumulate less lipids [38], and no changes in BAT weight, apart from larger mitochondrial size, were observed in KD-fed mice [71]. The lack of SIRT1 changes in BAT suggest that, under ketogenic stimulation, SIRT1 exerts its activity predominantly in white fat. However, the role of SIRT1 in BAT is highly debated, and some authors reported the UCP1 increase in BAT during ketosis [72], or in conjunction with the activation of the β3-adrenergic receptors [73]; whereas, others identified SIRT1 as the main protection from insulin resistance, and impaired β-adrenergic responses triggered by BAT inflammation. [74]. Further dedicated studies are needed to evaluate the effect of ketosis on SIRT1 in BAT.

From a clinical point of view, SIRT1 leads the balance from energy storage to energy expenditure, controlling fatty acid oxidation, and being substantially involved in the prevention of fat dysregulation and metabolic disorder development, highlighting the importance of its expression in adipose tissue to ensure fat mass health [75]. In this regard, SIRT1 is significantly higher in the SAT of normal-weight subjects than in obese or prediabetic subjects [18], and the reduction of SIRT1 expression in VAT promotes the potential for accumulation of ectopic fat [41]. In our experiments, ketosis did not change the weight of adipose tissue depots. This is in line with findings reporting that the overexpression of SIRT1 does not affect adipogenesis in a metabolic state characterized by increased lipolysis, and reduced adipogenesis induced through the WAT modulation of SIRT1 [66].

We have shown that serum levels of SIRT1 increase significantly during a KD, consistently with those of βHB. Nevertheless, the availability of NAD+ resulting from a KD can exert a modulated activity of NAD+-dependent enzymes, including the NAD+ consumers’ SIRTs [60,76,77]. However, beyond the evidence that SIRT1 increases with CR, we observed that a KD induces SIRT1 expression despite being high in fat, suggesting that the activation of SIRT1 downstream pathways could be the origin of the broad beneficial effects of the KD, regardless of weight loss and obesogenic diet.

Accordingly, we found that blood glucose and triglycerides did not differ between KD and the control group. A KD has several proven benefits for the glyco-lipidic profile, and diabetic or dyslipidemic patients might improve with a KD [32,56]. Like ketosis, SIRT1 improves carbohydrate and lipid metabolism, protecting against high-fat diet-induced metabolic damage [78]. Mice with selective adipose tissue-disrupted SIRT1 activity show premature metabolic aging, characterized by hyperglycemia, dyslipidemia, insulin-resistance, and glucose intolerance [79]. The high-fat KD feeding resulted in liver steatosis. This was to be expected, since non-alcoholic fatty liver disease is commonly connected with obesity, insulin-resistance, and high-lipid diet intake [80,81]. It represents an ectopic fat deposition in a dysfunctional fat-storing liver. The long-term, high-fat KD for epileptic patients has been shown to induce liver steatosis and gallstone formation [82], but the beneficial effects of a low-calorie KD on liver steatosis are noticeable [83,84,85]. Weight loss usually causes a SIRT1 increase in the liver [39,61]. The SIRT1-transgenic mice livers show decreased triglyceride accumulation [38], and circulating SIRT1 is inversely associated with fat liver infiltration in obese patients [86]. In our study, SIRT1 protein expression was not modulated in the livers of ketogenic mice suggesting that, despite ketosis, the increased hepatic triglyceride content, due to the high-fat intake, does not allow for an increase in SIRT1.

KD exerts its effects through multiple molecular mechanisms, and one of those might possibly be the upregulation of SIRT1. Nevertheless, few studies examined the relationship between KBs and SIRT1 in blood and adipose tissue. A greater expression of SIRT1 in white fat depot could be required to prevent fat accumulation, and maximize the thermogenic capacity. The increase in SIRT1 induced by KD may represent an adipose tissue protective mechanism against inflammation, obesity, and insulin-resistance induced by a high-fat diet. Further studies are required to determine the mechanism by which KBs and SIRT1 enhance the quality and function of adipose tissue.

## 4. Materials and Methods

### 4.1. Animals and Dietary Treatment

For this study, 10-week-old C57BL6/J male mice (Charles River Laboratories, Wilmington, MA, USA) were housed at Policlinico Umberto I animal facility with free access to water and standard food under 12 h light/dark cycles. Animals received either a low-carbohydrate, high-fat ketogenic diet (KD) (Research Diets, D10070801, n = 10) or a control diet (NC) (Research Diets, D19082304, n = 8) for 4 weeks. The macronutrient compositions of the diets are specified in Table 1. The KD contained cocoa butter as the principal source of fat. The calorie intake of the two diets can be calculated as follows: 6.7 kcal/g for KD vs. 3.8 kcal/g for NC. Diets were provided ad libitum throughout experiments, and food and calorie intake were determined. Body weight was recorded before the diet switch, and once/week for the entire duration of treatment.

**Figure 7:**
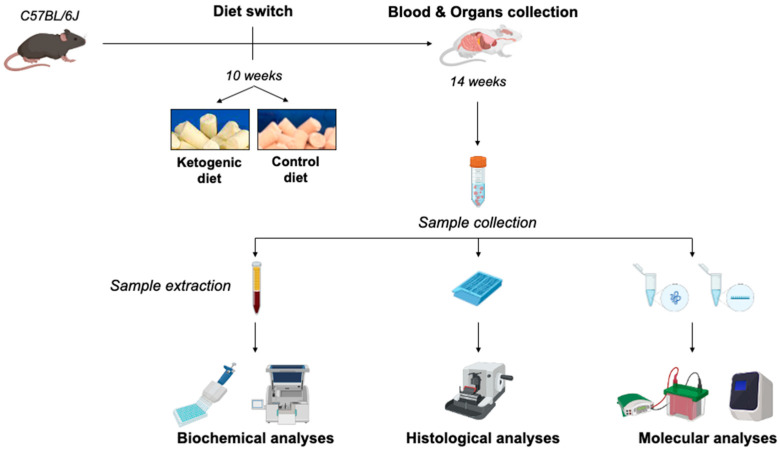
Schematic illustration of experimental design of the study.

At the end of dietary treatment animals were euthanized, and blood and organs were properly collected according to Figure 7. Steps were taken to minimize any suffering. All experiments were performed in accordance with Italian law (D.L. 2010/63EU) and the study was approved by the Sapienza University’s Animal Research Ethics Committee (OPBA MedMol/2019.01), and by the Italian Ministry of Health (n. 130/2020-PR).

### 4.2. Biochemical Analyses

#### 4.2.1. β-Hydroxybutyrate Assay

βHB levels were measured using a colorimetric assay kit (Cayman Chemicals, Ann Arbor, MI, USA) according to manufacturer’s instructions. Serum samples were filtered through 10kDa MWCO spin filters (Sigma Aldrich, St. Louis, MO, USA) before assaying. Absorbance (450 nm) was recorded using a D3 Plate Reader (DAS, Palombara Sabina, Italy). A standard curve was generated using MyCurveFit Data Analysis Tool (My Assays Ltd., Birmingham, UK), and each sample was assayed in triplicates.

#### 4.2.2. SIRT1 Assay

SIRT1 serum levels were measured using a colorimetric assay kit (Cusabio Technologies, Houston, TX, USA) according to manufacturer’s instructions. Absorbance (450 nm) was recorded using a D3 Plate Reader (DAS, Italy). A standard curve was generated using MyCurveFit Data Analysis Tool (My Assays Ltd.), and each sample was assayed in triplicates.

#### 4.2.3. Metabolic Profile

Serum fasting glucose and triglycerides (TG) concentrations were determined using the Multicare In Strips System (BSI Diagnostics, Arezzo, Italy). Each sample was assayed in triplicates.

### 4.3. Histological Analysis

Livers were fixed in 10% neutral buffered Formalin (Sigma-Aldrich, Burlington, MA, USA), dehydrated with increased grade alcohols, and embedded in Paraffin (Bio Optica, Milano, Italy). Five-micrometer sections obtained with the HM355S Microtome (Thermo Fisher Scientific, Waltham, MA, USA) were de-waxed, re-hydrated, and finally stained with Hematoxylin and Eosin (Sigma-Aldrich, Burlington, MA, USA). Slides were mounted and images were acquired using a Zeiss Axiovert 200 inverted microscope (Carl Zeiss, Inc., White Plains, NY, USA) equipped with a AxioCam 503 Color (Carl Zeiss, Inc., White Plains, NY, USA), and a 10X PanFluor Objective (Carl Zeiss, Inc., White Plains, NY, USA).

### 4.4. Molecular Analyses

Western blotting analysis of SIRT1 was performed on lysates obtained from liver, perigonadal visceral adipose tissue (VAT), subcutaneous adipose tissue (SAT), and interscapular brown adipose tissue (BAT) biopsies. Tissues were lysed in 50 mM Tris–HCl pH 7.4, 150 mM NaCl, 1% *w/v* NP-40, 0.25% *w/v* sodium deoxycholate, 1 mM EDTA, 0.5 *v/v* mM dithiothreitol, 10 mM β-glycerophosphate, 0.1 mM sodium vanadate, and protease inhibitor cocktail (Sigma-Aldrich, Burlington, MA, USA). SDS containing a sample buffer was added to lysates, and samples were boiled for 5 min at 95 °C. Denatured samples were electrophoresed in polyacrylamide gels, and transferred onto (Amersham, New York, NY, USA) membranes. Primary antibodies were incubated overnight at 4 °C, and secondary antibodies were incubated for 1 hour at room temperature. Blots were probed with rabbit polyclonal SIRT1 (Abcam, Cambridge, UK) and rabbit polyclonal β-ACTIN (Sigma-Aldrich, Burlington, MA, USA) antibodies. Signals were detected with horseradish peroxidase (HRP)-conjugated secondary antibodies, and enhanced chemiluminescence (Thermo Fisher Scientific, Waltham, MA, USA). Chemiluminescent images of immunodetected bands were recorded with the Syngene G-box system (Syngene Bioimaging, Syngene, Frederick, MD, USA), and immunoblot intensities were quantitatively analyzed using ImageJ Software (NIH, Bethesda, MD, USA). Results represent the means of at least three independent experiments, and were normalized to the amount of housekeeping proteins.

### 4.5. Statistical Analysis

Data obtained are presented as mean ± SEM from at least three independent experiments. The significance of the data was analyzed using the Student’s *t*-test for parametric data, and the Mann–Whitney test with Bonferroni corrections for nonparametric data. Correlation analyses were performed using linear regression. *p*  <  0.05 was considered statistically significant (* *p*  <  0.05, ** *p*  <  0.01, *** *p*  <  0.005, **** *p*  <  0.001). All analyses were performed using GraphPad Prism 7 software and SPSS.

## 5. Conclusions

Our data indicate that nutritional ketosis improves the metabolic status of adipose tissue. A KD exerts profound effects on SIRT1 expression in WAT, counteracting high fat-induced obesogenic stimuli. The beneficial effects of ketosis may depend, at least in part, on SIRT1, and the significance of a synergistic role between KBs and SIRT1 points to these bioactive molecules as valuable tools in the management of fat excess-related diseases.

## Figures and Tables

**Table 1:** Diet composition.

Component	Ketogenic Diet (#D10070801)		Control Diet (#D19082304)	
	g%	kcal%	g%	kcal%
Protein	16.8	10	9.6	10
Carbohydrate	0.2	0.1	77	80
Fat	67	90	4.3	10
Total		100		100
Kcal/g	6.7		3.8	

## Data Availability

All relevant data are included in this article. Data are available upon request to the corresponding author.

## References

[1] Obesity and Overweight. https://www.who.int/news-room/fact-sheets/detail/obesity-and-overweight.

[2] Ward Z.J., Bleich S.N., Cradock A.L., Barrett J.L., Giles C.M., Flax C., Long M.W., Gortmaker S.L. (2019). Projected U.S. State-Level Prevalence of Adult Obesity and Severe Obesity. N. Engl. J. Med..

[3] Tozzi R., Cipriani F., Masi D., Basciani S., Watanabe M., Lubrano C., Gnessi L., Mariani S. (2022). Ketone Bodies and SIRT1, Synergic Epigenetic Regulators for Metabolic Health: A Narrative Review. Nutrients.

[4] Crujeiras A.B., Izquierdo A.G., Primo D., Milagro F.I., Sajoux I., Jácome A., Fernandez-Quintela A., Portillo M.P., Martínez J.A., Martinez-Olmos M.A. (2021). Epigenetic Landscape in Blood Leukocytes Following Ketosis and Weight Loss Induced by a Very Low Calorie Ketogenic Diet (VLCKD) in Patients with Obesity. Clin. Nutr..

[5] Puchalska P., Crawford P.A. (2017). Multi-Dimensional Roles of Ketone Bodies in Fuel Metabolism, Signaling, and Therapeutics. Cell Metab..

[6] Kovács Z., Brunner B., Ari C. (2021). Beneficial Effects of Exogenous Ketogenic Supplements on Aging Processes and Age-Related Neurodegenerative Diseases. Nutrients.

[7] Newman J.C., Covarrubias A.J., Zhao M., Yu X., Gut P., Ng C.-P., Huang Y., Haldar S., Verdin E. (2017). Ketogenic Diet Reduces Midlife Mortality and Improves Memory in Aging Mice. Cell Metab..

[8] Roberts M.N., Wallace M.A., Tomilov A.A., Zhou Z., Marcotte G.R., Tran D., Perez G., Gutierrez-Casado E., Koike S., Knotts T.A. (2017). A Ketogenic Diet Extends Longevity and Healthspan in Adult Mice. Cell Metab..

[9] Castellana M., Conte E., Cignarelli A., Perrini S., Giustina A., Giovanella L., Giorgino F., Trimboli P. (2020). Efficacy and Safety of Very Low Calorie Ketogenic Diet (VLCKD) in Patients with Overweight and Obesity: A Systematic Review and Meta-Analysis. Rev. Endocr. Metab. Disord..

[10] Dabke P., Das A.M. (2020). Mechanism of Action of Ketogenic Diet Treatment: Impact of Decanoic Acid and Beta-Hydroxybutyrate on Sirtuins and Energy Metabolism in Hippocampal Murine Neurons. Nutrients.

[11] Caprio M., Infante M., Moriconi E., Armani A., Fabbri A., Mantovani G., Mariani S., Lubrano C., Poggiogalle E., Migliaccio S. (2019). Very-Low-Calorie Ketogenic Diet (VLCKD) in the Management of Metabolic Diseases: Systematic Review and Consensus Statement from the Italian Society of Endocrinology (SIE). J. Endocrinol. Investig..

[12] Barrea L., Caprio M., Camajani E., Verde L., Elce A., Frias-Toral E., Ceriani F., Cucalón G., Garcia-Velasquez E., El Ghoch M. (2022). Clinical and Nutritional Management of Very-Low-Calorie Ketogenic Diet (VLCKD) in Patients with Psoriasis and Obesity: A Practical Guide for the Nutritionist. Crit. Rev. Food Sci. Nutr..

[13] Guarente L. (2016). CELL METABOLISM. The Resurgence of NAD^+^. Science.

[14] Imai S., Armstrong C.M., Kaeberlein M., Guarente L. (2000). Transcriptional Silencing and Longevity Protein Sir2 Is an NAD-Dependent Histone Deacetylase. Nature.

[15] Herranz D., Muñoz-Martin M., Cañamero M., Mulero F., Martinez-Pastor B., Fernandez-Capetillo O., Serrano M. (2010). Sirt1 Improves Healthy Ageing and Protects from Metabolic Syndrome-Associated Cancer. Nat. Commun..

[16] Bordone L., Guarente L. (2005). Calorie Restriction, SIRT1 and Metabolism: Understanding Longevity. Nat. Rev. Mol. Cell Biol..

[17] Sassone-Corsi P. (2013). Physiology. When Metabolism and Epigenetics Converge. Science.

[18] Song Y.S., Lee S.K., Jang Y.J., Park H.S., Kim J.-H., Lee Y.J., Heo Y.-S. (2013). Association between Low SIRT1 Expression in Visceral and Subcutaneous Adipose Tissues and Metabolic Abnormalities in Women with Obesity and Type 2 Diabetes. Diabetes Res. Clin. Pract..

[19] Mariani S., Fiore D., Persichetti A., Basciani S., Lubrano C., Poggiogalle E., Genco A., Donini L.M., Gnessi L. (2016). Circulating SIRT1 Increases After Intragastric Balloon Fat Loss in Obese Patients. Obes. Surg..

[20] Mariani S., di Giorgio M.R., Martini P., Persichetti A., Barbaro G., Basciani S., Contini S., Poggiogalle E., Sarnicola A., Genco A. (2018). Inverse Association of Circulating SIRT1 and Adiposity: A Study on Underweight, Normal Weight, and Obese Patients. Front. Endocrinol..

[21] Sosnowska B., Mazidi M., Penson P., Gluba-Brzózka A., Rysz J., Banach M. (2017). The Sirtuin Family Members SIRT1, SIRT3 and SIRT6: Their Role in Vascular Biology and Atherogenesis. Atherosclerosis.

[22] Li Y., Wong K., Giles A., Jiang J., Lee J.W., Adams A.C., Kharitonenkov A., Yang Q., Gao B., Guarente L. (2014). Hepatic SIRT1 Attenuates Hepatic Steatosis and Controls Energy Balance in Mice by Inducing Fibroblast Growth Factor 21. Gastroenterology.

[23] Ren R., Wang Z., Wu M., Wang H. (2020). Emerging Roles of SIRT1 in Alcoholic Liver Disease. Int. J. Biol. Sci..

[24] Wang W., Sun W., Cheng Y., Xu Z., Cai L. (2019). Role of Sirtuin-1 in Diabetic Nephropathy. J. Mol. Med..

[25] Waldman M., Cohen K., Yadin D., Nudelman V., Gorfil D., Laniado-Schwartzman M., Kornwoski R., Aravot D., Abraham N.G., Arad M. (2018). Regulation of Diabetic Cardiomyopathy by Caloric Restriction Is Mediated by Intracellular Signaling Pathways Involving “SIRT1 and PGC-1α”. Cardiovasc. Diabetol..

[26] Cohen-Kfir E., Artsi H., Levin A., Abramowitz E., Bajayo A., Gurt I., Zhong L., D’Urso A., Toiber D., Mostoslavsky R. (2011). Sirt1 Is a Regulator of Bone Mass and a Repressor of Sost Encoding for Sclerostin, a Bone Formation Inhibitor. Endocrinology.

[27] Tozzi R., Masi D., Cipriani F., Contini S., Gangitano E., Spoltore M.E., Barchetta I., Basciani S., Watanabe M., Baldini E. (2022). Circulating SIRT1 and Sclerostin Correlates with Bone Status in Young Women with Different Degrees of Adiposity. Nutrients.

[28] Nakagawa T., Guarente L. (2011). Sirtuins at a Glance. J. Cell Sci..

[29] Abduraman M.A., Azizan N.A., Teoh S.H., Tan M.L. (2021). Ketogenesis and SIRT1 as a Tool in Managing Obesity. Obes. Res. Clin. Pract..

[30] He M.-Q., Wang J.-Y., Wang Y., Sui J., Zhang M., Ding X., Zhao Y., Chen Z.-Y., Ren X.-X., Shi B.-Y. (2020). High-Fat Diet-Induced Adipose Tissue Expansion Occurs Prior to Insulin Resistance in C57BL/6J Mice. Chronic Dis. Transl. Med..

[31] Campolo F., Catanzaro G., Venneri M.A., Ferretti E., Besharat Z.M. (2022). MicroRNA Loaded Edible Nanoparticles: An Emerging Personalized Therapeutic Approach for the Treatment of Obesity and Metabolic Disorders. Theranostics.

[32] Basciani S., Costantini D., Contini S., Persichetti A., Watanabe M., Mariani S., Lubrano C., Spera G., Lenzi A., Gnessi L. (2015). Safety and Efficacy of a Multiphase Dietetic Protocol with Meal Replacements Including a Step with Very Low Calorie Diet. Endocrine.

[33] Peña-Romero A.C., Navas-Carrillo D., Marín F., Orenes-Piñero E. (2018). The Future of Nutrition: Nutrigenomics and Nutrigenetics in Obesity and Cardiovascular Diseases. Crit. Rev. Food Sci. Nutr..

[34] Smith R.L., Soeters M.R., Wüst R.C.I., Houtkooper R.H. (2018). Metabolic Flexibility as an Adaptation to Energy Resources and Requirements in Health and Disease. Endocr. Rev..

[35] Cinti S. (2018). Adipose Organ Development and Remodeling. Compr. Physiol..

[36] Mariani S., Di Giorgio M.R., Rossi E., Tozzi R., Contini S., Bauleo L., Cipriani F., Toscano R., Basciani S., Barbaro G. (2020). Blood SIRT1 Shows a Coherent Association with Leptin and Adiponectin in Relation to the Degree and Distribution of Adiposity: A Study in Obesity, Normal Weight and Anorexia Nervosa. Nutrients.

[37] Cohen H.Y., Miller C., Bitterman K.J., Wall N.R., Hekking B., Kessler B., Howitz K.T., Gorospe M., de Cabo R., Sinclair D.A. (2004). Calorie Restriction Promotes Mammalian Cell Survival by Inducing the SIRT1 Deacetylase. Science.

[38] Pardo R., Velilla M., Herrero L., Cervela L., Ribeiro M.L., Simó R., Villena J.A. (2021). Calorie Restriction and SIRT1 Overexpression Induce Different Gene Expression Profiles in White Adipose Tissue in Association with Metabolic Improvement. Mol. Nutr. Food Res..

[39] Chen D., Bruno J., Easlon E., Lin S.-J., Cheng H.-L., Alt F.W., Guarente L. (2008). Tissue-Specific Regulation of SIRT1 by Calorie Restriction. Genes Dev..

[40] Rong Y.-L., Pan Y.-M., Huang J.-J., Yu C., Zhu K.-Y., Chen M.-L. (2020). Expression of Sirtuin 1 in visceral adipose tissue in Tibetan mini-pigs with obesity and insulin resistance induced by high fat/cholesterol diet. Zhongguo Ying Yong Sheng Li Xue Za Zhi.

[41] Perrini S., Porro S., Nigro P., Cignarelli A., Caccioppoli C., Genchi V.A., Martines G., De Fazio M., Capuano P., Natalicchio A. (2020). Reduced SIRT1 and SIRT2 Expression Promotes Adipogenesis of Human Visceral Adipose Stem Cells and Associates with Accumulation of Visceral Fat in Human Obesity. Int. J. Obes..

[42] Mariani S., Di Rocco G., Toietta G., Russo M.A., Petrangeli E., Salvatori L. (2017). Sirtuins 1-7 Expression in Human Adipose-Derived Stem Cells from Subcutaneous and Visceral Fat Depots: Influence of Obesity and Hypoxia. Endocrine.

[43] Costa C.d.S., Hammes T.O., Rohden F., Margis R., Bortolotto J.W., Padoin A.V., Mottin C.C., Guaragna R.M. (2010). SIRT1 Transcription Is Decreased in Visceral Adipose Tissue of Morbidly Obese Patients with Severe Hepatic Steatosis. Obes. Surg..

[44] Masi D., Spoltore M.E., Rossetti R., Watanabe M., Tozzi R., Caputi A., Risi R., Balena A., Gandini O., Mariani S. (2022). The Influence of Ketone Bodies on Circadian Processes Regarding Appetite, Sleep and Hormone Release: A Systematic Review of the Literature. Nutrients.

[45] Wątroba M., Dudek I., Skoda M., Stangret A., Rzodkiewicz P., Szukiewicz D. (2017). Sirtuins, Epigenetics and Longevity. Ageing Res. Rev..

[46] Kolb H., Kempf K., Röhling M., Lenzen-Schulte M., Schloot N.C., Martin S. (2021). Ketone Bodies: From Enemy to Friend and Guardian Angel. BMC Med..

[47] Ruderman N.B., Xu X.J., Nelson L., Cacicedo J.M., Saha A.K., Lan F., Ido Y. (2010). AMPK and SIRT1: A Long-Standing Partnership?. Am. J. Physiol. Endocrinol. Metab..

[48] Cantó C., Gerhart-Hines Z., Feige J.N., Lagouge M., Noriega L., Milne J.C., Elliott P.J., Puigserver P., Auwerx J. (2009). AMPK Regulates Energy Expenditure by Modulating NAD+ Metabolism and SIRT1 Activity. Nature.

[49] Liou C.-J., Lee Y.-K., Ting N.-C., Chen Y.-L., Shen S.-C., Wu S.-J., Huang W.-C. (2019). Protective Effects of Licochalcone A Ameliorates Obesity and Non-Alcoholic Fatty Liver Disease Via Promotion of the Sirt-1/AMPK Pathway in Mice Fed a High-Fat Diet. Cells.

[50] Price N.L., Gomes A.P., Ling A.J.Y., Duarte F.V., Martin-Montalvo A., North B.J., Agarwal B., Ye L., Ramadori G., Teodoro J.S. (2012). SIRT1 Is Required for AMPK Activation and the Beneficial Effects of Resveratrol on Mitochondrial Function. Cell Metab..

[51] Xie Z., Zhang D., Chung D., Tang Z., Huang H., Dai L., Qi S., Li J., Colak G., Chen Y. (2016). Metabolic Regulation of Gene Expression by Histone Lysine β-Hydroxybutyrylation. Mol. Cell.

[52] Xu Y., Yu T., Ma G., Zheng L., Jiang X., Yang F., Wang Z., Li N., He Z., Song X. (2021). Berberine Modulates Deacetylation of PPARγ to Promote Adipose Tissue Remodeling and Thermogenesis via AMPK/SIRT1 Pathway. Int. J. Biol. Sci..

[53] Kang Y.-M., Kang H.-A., Cominguez D.C., Kim S.-H., An H.-J. (2021). Papain Ameliorates Lipid Accumulation and Inflammation in High-Fat Diet-Induced Obesity Mice and 3T3-L1 Adipocytes via AMPK Activation. Int. J. Mol. Sci..

[54] Zhou T., Cheng X., He Y., Xie Y., Xu F., Xu Y., Huang W. (2022). Function and Mechanism of Histone β-Hydroxybutyrylation in Health and Disease. Front. Immunol..

[55] Nishitani S., Fukuhara A., Shin J., Okuno Y., Otsuki M., Shimomura I. (2018). Metabolomic and Microarray Analyses of Adipose Tissue of Dapagliflozin-Treated Mice, and Effects of 3-Hydroxybutyrate on Induction of Adiponectin in Adipocytes. Sci. Rep..

[56] Yuan X., Wang J., Yang S., Gao M., Cao L., Li X., Hong D., Tian S., Sun C. (2020). Effect of the Ketogenic Diet on Glycemic Control, Insulin Resistance, and Lipid Metabolism in Patients with T2DM: A Systematic Review and Meta-Analysis. Nutr. Diabetes.

[57] Ciaffi J., Mitselman D., Mancarella L., Brusi V., Lisi L., Ruscitti P., Cipriani P., Meliconi R., Giacomelli R., Borghi C. (2021). The Effect of Ketogenic Diet on Inflammatory Arthritis and Cardiovascular Health in Rheumatic Conditions: A Mini Review. Front. Med..

[58] Schiavo L., Pierro R., Asteria C., Calabrese P., Di Biasio A., Coluzzi I., Severino L., Giovanelli A., Pilone V., Silecchia G. (2022). Low-Calorie Ketogenic Diet with Continuous Positive Airway Pressure to Alleviate Severe Obstructive Sleep Apnea Syndrome in Patients with Obesity Scheduled for Bariatric/Metabolic Surgery: A Pilot, Prospective, Randomized Multicenter Comparative Study. Obes. Surg..

[59] Muscogiuri G., Barrea L., Campolo F., Sbardella E., Sciammarella C., Tarsitano M.G., Bottiglieri F., Colao A., Faggiano A. (2022). NIKE Ketogenic Diet: A Tool for the Management of Neuroendocrine Neoplasms?. Crit. Rev. Food Sci. Nutr..

[60] Wei X., Jia R., Wang G., Hong S., Song L., Sun B., Chen K., Wang N., Wang Q., Luo X. (2020). Depot-Specific Regulation of NAD+/SIRTs Metabolism Identified in Adipose Tissue of Mice in Response to High-Fat Diet Feeding or Calorie Restriction. J. Nutr. Biochem..

[61] Moschen A.R., Wieser V., Gerner R.R., Bichler A., Enrich B., Moser P., Ebenbichler C.F., Kaser S., Tilg H. (2013). Adipose Tissue and Liver Expression of SIRT1, 3, and 6 Increase after Extensive Weight Loss in Morbid Obesity. J. Hepatol..

[62] Bachmanov A.A., Reed D.R., Tordoff M.G., Price R.A., Beauchamp G.K. (2001). Nutrient Preference and Diet-Induced Adiposity in C57BL/6ByJ and 129P3/J Mice. Physiol. Behav..

[63] Lewis S.R., Ahmed S., Dym C., Khaimova E., Kest B., Bodnar R.J. (2005). Inbred Mouse Strain Survey of Sucrose Intake. Physiol. Behav..

[64] Giralt M., Villarroya F. (2013). White, Brown, Beige/Brite: Different Adipose Cells for Different Functions?. Endocrinology.

[65] Rodríguez A., Becerril S., Hernández-Pardos A.W., Frühbeck G. (2020). Adipose Tissue Depot Differences in Adipokines and Effects on Skeletal and Cardiac Muscle. Curr. Opin. Pharmacol..

[66] Qiang L., Wang L., Kon N., Zhao W., Lee S., Zhang Y., Rosenbaum M., Zhao Y., Gu W., Farmer S.R. (2012). Brown Remodeling of White Adipose Tissue by SirT1-Dependent Deacetylation of Pparγ. Cell.

[67] Li X. (2013). SIRT1 and Energy Metabolism. Acta Biochim. Biophys. Sin..

[68] Timmons J.A., Wennmalm K., Larsson O., Walden T.B., Lassmann T., Petrovic N., Hamilton D.L., Gimeno R.E., Wahlestedt C., Baar K. (2007). Myogenic Gene Expression Signature Establishes That Brown and White Adipocytes Originate from Distinct Cell Lineages. Proc. Natl. Acad. Sci. USA.

[69] Wang H., Chen Y., Mao X., Du M. (2019). Maternal Obesity Impairs Fetal Mitochondriogenesis and Brown Adipose Tissue Development Partially via Upregulation of MiR-204-5p. Biochim. Biophys. Acta Mol. Basis Dis..

[70] Picard F., Kurtev M., Chung N., Topark-Ngarm A., Senawong T., Machado De Oliveira R., Leid M., McBurney M.W., Guarente L. (2004). Sirt1 Promotes Fat Mobilization in White Adipocytes by Repressing PPAR-Gamma. Nature.

[71] Kennedy A.R., Pissios P., Otu H., Xue B., Asakura K., Furukawa N., Marino F.E., Liu F.-F., Kahn B.B., Libermann T.A. (2007). A High-Fat, Ketogenic Diet Induces a Unique Metabolic State in Mice. Am. J. Physiol.-Endocrinol. Metab..

[72] Srivastava S., Baxa U., Niu G., Chen X., Veech R.L. (2013). A Ketogenic Diet Increases Brown Adipose Tissue Mitochondrial Proteins and UCP1 Levels in Mice. IUBMB Life.

[73] Douris N., Desai B.N., Fisher F.M., Cisu T., Fowler A.J., Zarebidaki E., Nguyen N.L.T., Morgan D.A., Bartness T.J., Rahmouni K. (2017). Beta-Adrenergic Receptors Are Critical for Weight Loss but Not for Other Metabolic Adaptations to the Consumption of a Ketogenic Diet in Male Mice. Mol. Metab..

[74] Escalona-Garrido C., Vázquez P., Mera P., Zagmutt S., García-Casarrubios E., Montero-Pedrazuela A., Rey-Stolle F., Guadaño-Ferraz A., Rupérez F.J., Serra D. (2020). Moderate SIRT1 Overexpression Protects against Brown Adipose Tissue Inflammation. Mol. Metab..

[75] Thyagarajan B., Foster M.T. (2017). Beiging of White Adipose Tissue as a Therapeutic Strategy for Weight Loss in Humans. Horm. Mol. Biol. Clin. Investig..

[76] Elamin M., Ruskin D.N., Masino S.A., Sacchetti P. (2018). Ketogenic Diet Modulates NAD+-Dependent Enzymes and Reduces DNA Damage in Hippocampus. Front. Cell. Neurosci..

[77] Cambronne X.A., Stewart M.L., Kim D., Jones-Brunette A.M., Morgan R.K., Farrens D.L., Cohen M.S., Goodman R.H. (2016). Biosensor Reveals Multiple Sources for Mitochondrial NAD^+^. Science.

[78] Pfluger P.T., Herranz D., Velasco-Miguel S., Serrano M., Tschöp M.H. (2008). Sirt1 Protects against High-Fat Diet-Induced Metabolic Damage. Proc. Natl. Acad. Sci. USA.

[79] Xu C., Cai Y., Fan P., Bai B., Chen J., Deng H.-B., Che C.-M., Xu A., Vanhoutte P.M., Wang Y. (2015). Calorie Restriction Prevents Metabolic Aging Caused by Abnormal SIRT1 Function in Adipose Tissues. Diabetes.

[80] Recena Aydos L., Aparecida do Amaral L., Serafim de Souza R., Jacobowski A.C., Freitas Dos Santos E., Rodrigues Macedo M.L. (2019). Nonalcoholic Fatty Liver Disease Induced by High-Fat Diet in C57bl/6 Models. Nutrients.

[81] Lian C.-Y., Zhai Z.-Z., Li Z.-F., Wang L. (2020). High Fat Diet-Triggered Non-Alcoholic Fatty Liver Disease: A Review of Proposed Mechanisms. Chem. Biol. Interact..

[82] Arslan N., Guzel O., Kose E., Yılmaz U., Kuyum P., Aksoy B., Çalık T. (2016). Is Ketogenic Diet Treatment Hepatotoxic for Children with Intractable Epilepsy?. Seizure.

[83] Watanabe M., Tozzi R., Risi R., Tuccinardi D., Mariani S., Basciani S., Spera G., Lubrano C., Gnessi L. (2020). Beneficial Effects of the Ketogenic Diet on Nonalcoholic Fatty Liver Disease: A Comprehensive Review of the Literature. Obes. Rev..

[84] Risi R., Tozzi R., Watanabe M. (2021). Beyond Weight Loss in Nonalcoholic Fatty Liver Disease: The Role of Carbohydrate Restriction. Curr. Opin. Clin. Nutr. Metab. Care.

[85] Cunha G.M., Guzman G., Correa De Mello L.L., Trein B., Spina L., Bussade I., Marques Prata J., Sajoux I., Countinho W. (2020). Efficacy of a 2-Month Very Low-Calorie Ketogenic Diet (VLCKD) Compared to a Standard Low-Calorie Diet in Reducing Visceral and Liver Fat Accumulation in Patients With Obesity. Front. Endocrinol..

[86] Mariani S., Fiore D., Basciani S., Persichetti A., Contini S., Lubrano C., Salvatori L., Lenzi A., Gnessi L. (2015). Plasma Levels of SIRT1 Associate with Non-Alcoholic Fatty Liver Disease in Obese Patients. Endocrine.

